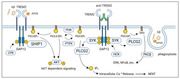# Generation and validation of anti‐TREM2 agonistic antibodies to enable the advancement of drug targets in the TREM2/DAP12 signaling pathway for the treatment of Alzheimer Disease

**DOI:** 10.1002/alz.088509

**Published:** 2025-01-09

**Authors:** Mustapha Moussaif, June Javens‐Wolfe, Alan D. Palkowitz, Timothy I. Richardson

**Affiliations:** ^1^ Indiana Biosciences Research Institute, Indianapolis, IN USA; ^2^ Indiana University School of Medicine, Indianapolis, IN USA

## Abstract

**Background:**

TREM2 signaling has been implicated in Alzheimer’s Disease (AD). TREM2 regulates microglial states and functions such as phagocytosis. The most prominent TREM signaling adapter is DAP12, encoded by TYROBP. Understanding functional changes of this complex, and downstream effectors such as SHIP1, PLCG2 and the Scr family kinases Lyn and Hck, is required to evaluate a broad range of therapeutic hypotheses and drug targets for prioritization and enablement. The lack of available, well validated, and openly distributed experimental tools can limit early drug discovery efforts. Therefore, the IUSM Purdue TREAT‐AD Center has generated and validated TREM2 activating antibodies to enable the advancement of drug targets in the TREM2/DAP12 signaling pathway.

**Method:**

To establish and validate anti‐TREM2 agonist antibodies, heavy and light chain variable sequences were identified from multiple publications including patent applications. Antibodies were formatted as either human IgG1, Fc null mutant IgG1 or antibody transport vehicle (ATV) Fc null mutant IgG1. They were expressed in mammalian ExpiCHO cells and tested *ex vivo* for agonism based on their ability to activate AKT and Syk phosphorylation in THP1 cells and TREM2/DAP12 overexpressing cells respectively. The strongest agonistic candidate was scaled, purified, and further characterized biophysically and functionally.

**Result:**

Several agonistic antibodies were identified. AL2p31 antibody showed binding specificity to human versus murine TREM2. Biophysical characterization using biolayer interferometry showed that binding kinetic parameters (KD, Kon, and Koff) were not significantly affected in LALAPG null mutant Fc background. AL2p31 specifically induced Syk phosphorylation in comparison to an isotype control. Analysis of antibodies formatted as bispecific IgG1 targeting both TREM2 and the human transferrin receptor (hTfR), confirmed that RS9‐F6 can bind both human and murine TREM2 and revealed the ATV 35‐21‐16 variant sequence as a binder for the hTfR.

**Conclusion:**

The mission of the IUSM Purdue TREAT‐AD Center is to enable and advance the next generation of drug targets for the treatment of AD. The validation of anti‐TREM2 agonistic antibodies as research tools will enable comprehensive studies of the TREM2/DAP12 signaling and potential drug targets within the pathway including SHIP1, PLCG2 and the Scr family kinase Lyn and Hck.